# Neutrality and Robustness in Evo-Devo: Emergence of Lateral Inhibition

**DOI:** 10.1371/journal.pcbi.1000226

**Published:** 2008-11-21

**Authors:** Andreea Munteanu, Ricard V. Solé

**Affiliations:** 1 ICREA-Complex Systems Lab, Universitat Pompeu Fabra (PRBB-GRIB), Barcelona, Spain; 2 Santa Fe Institute, Santa Fe, New Mexico, United States of America; University of Auckland, New Zealand

## Abstract

Embryonic development is defined by the hierarchical dynamical process that translates genetic information (genotype) into a spatial gene expression pattern (phenotype) providing the positional information for the correct unfolding of the organism. The nature and evolutionary implications of genotype–phenotype mapping still remain key topics in evolutionary developmental biology (evo-devo). We have explored here issues of neutrality, robustness, and diversity in evo-devo by means of a simple model of gene regulatory networks. The small size of the system allowed an exhaustive analysis of the entire fitness landscape and the extent of its neutrality. This analysis shows that evolution leads to a class of robust genetic networks with an expression pattern characteristic of lateral inhibition. This class is a repertoire of distinct implementations of this key developmental process, the diversity of which provides valuable clues about its underlying causal principles.

## Author Summary

The diversity of life is a consequence of changes in the genotype (genes and their interdependence), but it is upon the observable organism's morphology (phenotype) that natural selection acts. Thus, the study of genotype–phenotype mapping can reveal key mechanisms driving life's capacity of continuous evolution and resilience in diverse environments. In this context, it has been observed that small numbers of genes form robust functional developmental modules, hierarchically reused throughout development. Here we analyze the evolution of small genetic modules toward higher diversity and robustness. Given the small size of the gene network, we can afford to analyze all possible topologies and thus the entire fitness landscape. This exhaustive study as well as simulations of evolutionary processes uncover a set of genetic interactions producing robust and diverse phenotypes. We single out the distinctive features of these networks responsible for their stability against environmental and structural perturbations. More precisely, all these robust genotypes can be related to the key mechanism of lateral inhibition for which a cell of a given type inhibits its neighbors to keep them from adopting the same type. Their distinctive features can thus shed light on the underlying mechanisms leading to pattern formation through lateral inhibition.

## Introduction

The evolution of life forms on our planet has led to the generation of an enormous variety of living structures. How such patterns of organization emerge [1]–[3], how contingency [4] and constraints [5],[6] shape them and how they acquire robustness [7] are unanswered questions that have been at the forefront of biology for more than a century and are still open key questions. The research field encompassing these fundamental issues is referred to as evolutionary developmental biology or in short evo-devo [1]. With the increasing capacity of mathematical modeling to provide fresh insight into the biological processes [8], computer simulations and experimental approaches in this field have recently reached common ground (see [9],[10] as recent reviews).

A major conceptual problem for the modeling approach to evo-devo is the mapping between genotype (hereditary genetic information) and phenotype (the physical characteristics of the resultant organism). It is the phenotype that determines the organism's chances of survival (fitness), as it is on it that natural selection acts. The set of all genotypes, their resultant phenotypes and associated fitness is called fitness landscape. Since Wright's pioneering idea in the early 30's [11] that the hill-climbing process of population's adaptive evolution intimately depends on how smooth or rugged the fitness landscape is, numerous theoretical works have been contributing to what now can be considered as the theory of fitness landscapes [5], [12]–[14]. Moreover, empirical studies of fitness landscapes can nowadays be performed in the laboratory [15]–[17], revealing the real evolutionary paths undertaken by the organisms, and thus opening a previously-unavailable window on the actual evolution process.

The extensively studied theoretical case that has become the classic example of evolution in a fitness landscape is provided by RNA folding [18]–[20]. Here the genotype is defined by the nucleotide sequence, whereas the phenotype consists of the secondary structure formed by the (planar) pattern of the base pairs. Within the RNA context, the existence of iso-phenotypic genotypes (or neutrality) has significant implications in evolution, in general [21]–[24] and evo-devo, in particular [25]. More precisely, neutrality is hypothesized to allow a more exhaustive search in the genotype space and consequently, better accessibility to diverse and potentially fitter phenotypes [13],[26].

The neutrality feature has been encountered and studied in other works of similar nature to the RNA's, such as in the origin and complexification of the protein universe [27], or in tunable-neutrality models of abstract molecular species [28], but also in other fields of very different nature. An example is provided by a model of feed-forward signaling networks [29]. Here, a minimal Boolean network receives a set of input signals, and computes the output. The genotype is defined by the wiring diagram (the network topology plus the weight of each interaction), whereas the phenotype is specified by the Boolean computation being performed. An example closer to the current study is a Boolean model of genetic networks [30], a study that inquires on the requirement of “genetic flexibility” or more precisely, of phenotype continuity in evolution, and the subsequent constraints it may pose to species evolution in a changing environment. In a more recent work, the same group developed an evolutionary model of network evo-devo [31] that adds to the same approach as the current study, with the two works providing complementary clues on the evolution of minimal developmental modules. Again under the Boolean approach, Andreas Wagner's studies ranging from the “epigenetic stability” of developmental pathways [32] to bridging robustness and evolvability by means of neutrality features in models of gene networks [33] complete the framework in which the present work is formulated. Moreover, the present formulation constitutes a continuation of the model introduced in [34], as well as a Hawk's eye view of an isolated genetic sub-system. Its exhaustive study allows uncovering of features that are generally not accessible from statistical large-scale studies of similar nature. As far as we know, no parallel exhaustive analogies of Boolean approaches have been applied within the context of spatially-explicit evo-devo.

We have addressed here the role of neutrality and robustness in the evolution of minimal developmental modules. It is now apparent that the genetic networks responsible for major events in the development of organisms present significant robustness to a wide range of perturbations [35]. Moreover, experimental works reveal that certain genes and their interactions are recurrently encountered in very diverse organisms (e.g. Homeobox genes [1],[36]), suggesting that minimal genetic modules may underlie fundamental developmental pathways. The current work is inspired by the pioneering theoretical and empirical analysis of developmental genetic regulatory networks in long-germ-band insects (*Drosophila melanogaster*) ([37]–[39] and references therein) and plant (*Arabidopsis thaliana*) development [40]. As anticipated by [34], *Drosophila* is a suitable model organism to inquire on small gene modules that control specific parts of the development process. The goal of the current work is not a precise explanation of a specific genetic module, but a description of possible underlying principles of network assemblage and evolution.

In this context, our guiding questions are: what classes of spatial expression patterns can possibly emerge from signals mediated by juxtacrine (intra or inter-cellular) interactions in a minimal genetic network? Are there intrinsically robust modules and what are their defining characteristics? Our approach addressing these questions is organized as follows. We introduce the model of gene interactions whose dynamics provides the gene expression pattern. We present the minimal set of genes producing a specific, biologically-relevant expression pattern, and the exhaustive analysis of all possible gene interactions and their associated expression patterns. Among all these topologies, we identify those providing a robust expression pattern, being thus the candidates for the developmental modules discussed above. Ultimately, an evolutionary study of populations of such networks conditioned on diversity is presented, revealing rapid evolution towards robust stripe-like expression patterns. We show that the structure of the encountered minimal robust networks relates to the phenomenon of lateral inhibition, a widespread mechanism of biological pattern formation, emphasizing thus the importance of these minimal development-driving modules.

## Results

As mentioned in the introduction, the present work has as biological reference existent information on the logic of early development in two model systems: in long-germ-band insects and plants. For insects, during the *syncytium* phase, a series of chemical stripes forms, which are actually alternating evenly-spaced bands of transcription factors encoded by the *pair-rule* genes. These different cell states, defined also by the subsequent expression of the *segment polarity* genes, will determine the future body segments. The mechanisms responsible for the expression stripes have been the object of numerous studies, initiatives that have emphasized the necessity to uncover the gene circuitry or gene network topology [41]. Even though the importance of temporal and spatial expression of genes in development [42] has been addressed and demonstrated prior to the introduction of the gene circuit method [41],[43], only in the last decade has become apparent (also experimentally- and computationally-feasible) that the unification of topological, positional and dynamical information of gene expression is compulsory [44],[45].

In parallel with this unifying view on the mechanisms of stripe formation, the search for the underlying developmental bricks, the key driving interactions responsible for the robustness and accessibility of the segment polarity developmental pattern, has been the object of several studies [46],[47]. The same approach was successfully applied to uncover the structural robustness of the neurogenic gene network also in *Drosophila* embryo development [48]. In our modeling, we have approached the minimal module issue from a different perspective: on a more basic level of pattern formation mechanisms, on one hand, and on a more general level than the particularities of the segment polarity or neurogenic gene network, on the other hand. In so doing, we have searched for an organizing module of robust pattern formation within the features inspired from the observed modules in developmental biology.

### The Model

In the calculation of the expression patterns of the genetic networks, we have continued the Boolean approach of [34], and we have inspired also from more recent studies and extensions of the reaction-diffusion (continuous) connectionist model 49–52. By the existence of these two approaches, continuous and discrete (Boolean), or analog and digital, respectively, the resultant conclusions can pinpoint gradient-specific and topological mechanisms responsible for specific processes. In this sense, both approaches are needed and thus necessary for a complete understanding. The Boolean modeling approach has been widely employed in modeling the logic of genome architecture, of which development is a constitutive part [31],[34]. These models have been shown to successfully recover the same expression patterns as those resultant from continuous models [37],[53]. Even though we emphasize here the literature on Boolean modeling in evo-devo, the continuous approach of reaction-diffusion models constitutes the standard tool for evo-devo. Since the revolutionizing work of Alan Turing on pattern-formation and morphogenesis [54], there have been rapid and continuous advances in our understanding of what are now called Turing patterns (see [55] for a recent review). As in the case of Boolean modeling, this approach too is constantly employed for addressing new questions in this field. Until recently there has been a significant emphasis on the analyses providing answers to *how* gene networks work, an answer being mechanisms such as Turing bifurcations. With the advances in computational methods, the issue of increasing interest is *why* the gene networks have the topology observed, an issue that needs to be addressed in the light of evolution. Again, it is a problem whose resolution is facilitated by applying both approaches, continuous ([56],[57] just to mention a few) and discrete [31],[52].

**Figure 1:**
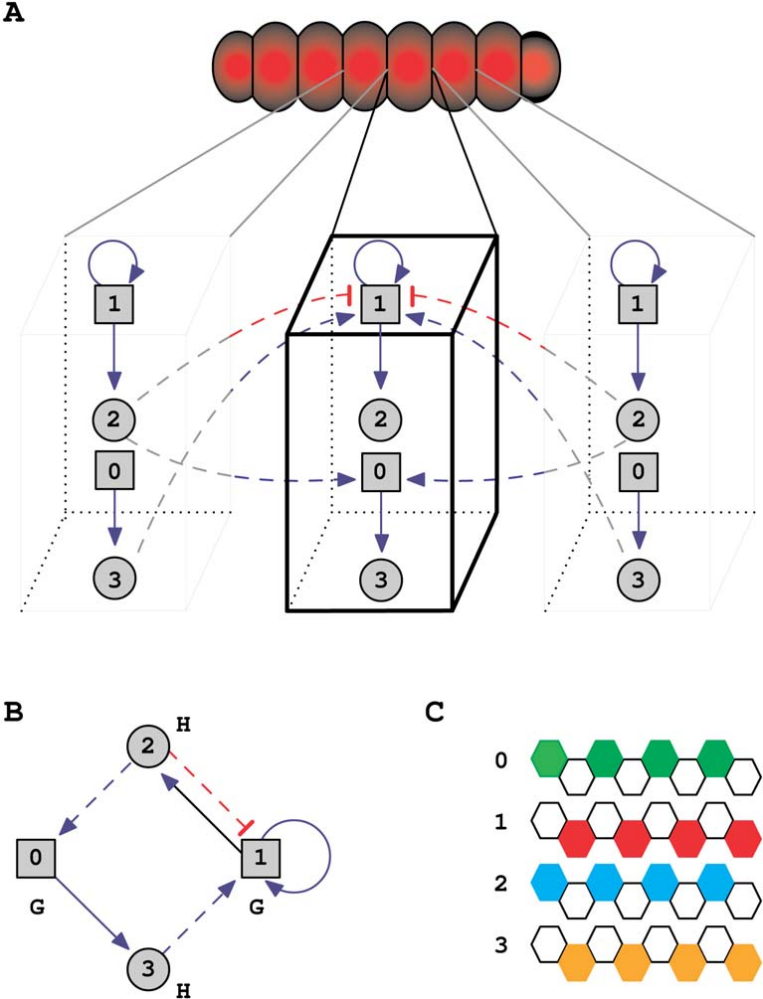
Illustration of the model's assumptions and structure. (A) Representation of the genetic interactions, with morphogenes coding for short-range signals (circles) affecting local genes (squares) only inter-cellularly (dashed links), and local genes interacting only intra-cellularly with all genes (solid links). (B) An example of an arbitrarily chosen interaction network (in panel *A* too), with the notation used throughout the work: red links–inhibition, blue links–activation. (C) The corresponding final gene expression of the 4 genes in the 8 cells, with white denoting inactive state.

In the current model, the network is composed of *N* genes whose state can be active (state = 1), or inactive (state = 0). Among these genes, a number *G* are *local genes* that code for intra-cellular molecules, and the rest *H*, are *hormones* [50] that code for short range, diffusible paracrine molecules (see Figure 1). More precisely, the first group of genes interact intra-cellularly with all the genes, while the short-range signaling proteins coded by hormones interact only inter-cellularly with the local genes, affecting thus their expression in neighboring cells. In the previous formulation of [34], the two types of interactions, local and non-local, are referred to as the internal and external gene network, respectively. In this context, a standard term in evo-devo for “hormone” is *morphogen* [58],[59], whose gradient concentration determines the fates of surrounding cells. Intimately related to the already mentioned concept of positional-information, the diffusion-controlled concentration and residence-time of a morphogen are interpreted by cells as committing signal for a certain state. We have chosen to employ here the term morphogen instead of hormone [50], even though our Boolean approach does not distinguish gradients of concentration.

**Figure 2:**
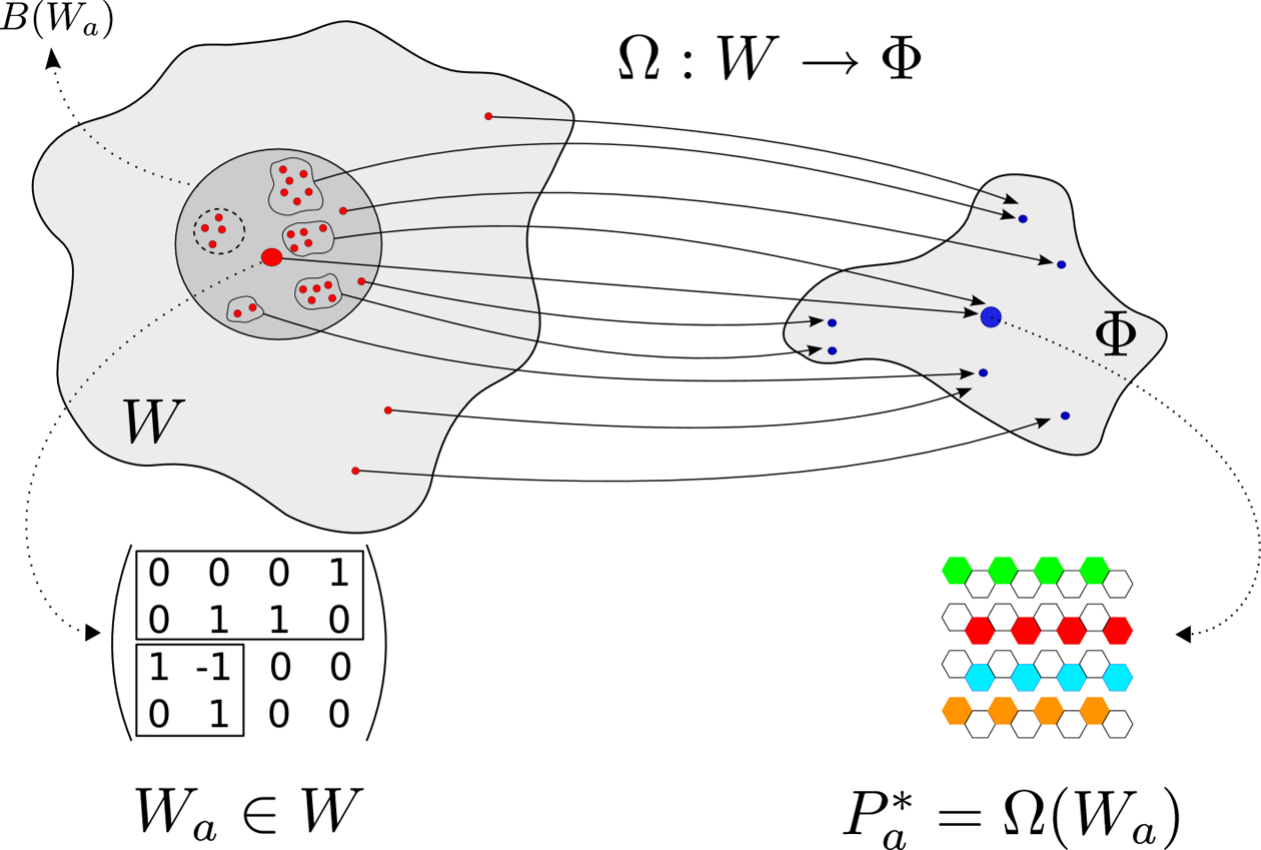
The genotype-phenotype mapping in the current model and for the case (*N*,*H*) = (4,2). The mapping from the wiring space *W* to the expression pattern space Φ. In the wiring matrix, the larger rectangle isolates the *G*×*N* matrix *A*, while the smaller square represents the *H*×*G* matrix *B*. The one-link mutants (gray circle *B*(*W_a_*) in *W*) of a genotype can relate to very diverse phenotypes.

We consider one-dimensional organisms composed of a collection of *C* cells. In our case, *C* = 8, with larger values having no substantial influence on the results presented here. The equations determining the time evolution of the pattern are
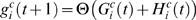
(1)

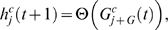
(2)where 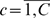 denotes cell index, 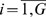, local gene's index, 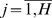, morphogen index, and
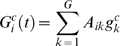
(3)

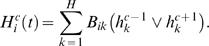
(4)Here the *G*×*N* matrix *A* (internal network; see also Figure 2) includes the intra-cellular interactions (continuous arrows in Figure 1) and the *H*×*G* matrix *B* (external network), the inter-cellular interactions (dashed arrows in Figure 1). The interactions consist of either activation or inhibition, with the values of the matrix being +1 or −1, respectively. The function ∨ is the “OR” function (the result is 1 if either of the short-range signals from neighboring cells is active, and 0, otherwise). For the two extremes of the organism (the anterior and posterior poles), the cells have a single neighbor. The function Θ is the threshold function yielding 1 if the argument is positive, and 0, otherwise.

As initial condition, a maternal signal is considered at the anterior pole (leftmost cell), with only the first gene being active, i.e. 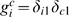; 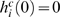, where *δ_ij_* = 1 if *i* = *j* and zero, otherwise. For this initial condition and the chosen interaction matrices, we determined the steady states. More precisely, we only consider the one-state attractors (fixed point attractor), discarding thus the unstable and the oscillatory cases (see Methods).

Using the previous definitions, we can define the mapping between wiring and pattern (Figure 2) as:
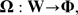
(5)implying that for each genotype (genes' wiring) *W_a_* = (*A_ij_*, *B_kl_*)∈**W**, we have a phenotype (expression pattern) 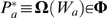. As we shall see, this system shares with other genotype-phenotype mappings a set of interesting features. On one hand, one-point mutants of a given genotype can generate very diverse phenotypes, and on the other, multiple genotypes can generate the same phenotype (Figure 2). One can also see that the Boolean approach allows a direct relationship between genotype and phenotype, a discretization that would have been hampered in a continuous modeling. As a first approach, we have studied the diversity of expression patterns with the aim of characterizing this genotype-phenotype mapping for a specific case of (*G*,*H*). Additionally, by introducing a fitness function, we have studied how adaptation proceeds through the nature of the mapping Ω.

### Networks and Pattern-Formation

In order to select a model for study, we have sought the existence of a specific expression-pattern feature that appears in all developmental modules studied so far. It consists in a stripe-like pattern of a one-cell-wide alternating active-inactive values. For the rules defined above, we found that the minimal number of genes capable of producing such an expression pattern is composed of 2 local genes and 2 morphogenes. Four-element networks have already been shown, through slightly different model assumptions, to be the minimal nets able to generate all possible types of Boolean spatial arrangements [52]. One can exhaustively study all the possible interaction networks of (*N*,*H*) = (4,2) as it is a tractable number: 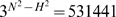. For larger networks, the number of configurations becomes intractable for an exhaustive study, but we shall address the statistical study of larger networks as a continuation of the present work.

**Figure 3:**
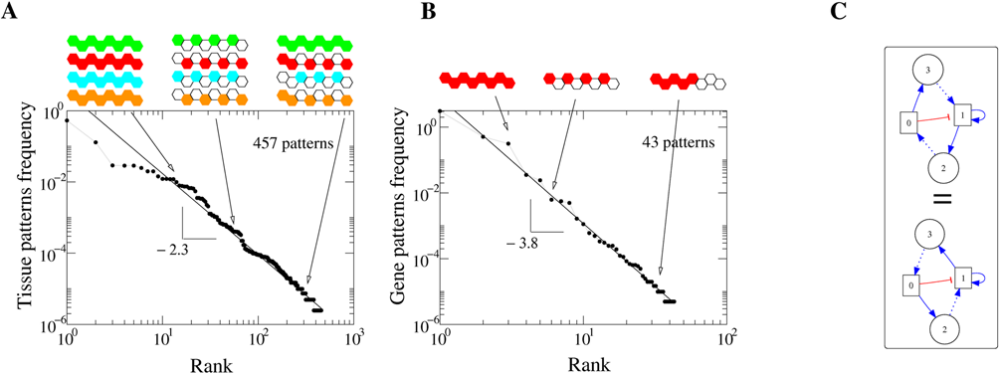
The diversity of patterns for the case (*N*,*H*) = (4,2). The frequency of organism (A) and (B) gene patterns ordered by rank. Also some examples of patterns are illustrated, where again white hexagons refer to inactive genes. The distribution of patterns follows *N*_1_(*r*)∝*r* ^−*γ*^, with *r* the rank, and *γ* = *γ_t_* = 2.3 for tissue patterns and *γ* = *γ_g_* = 3.8 for gene patterns. (C) An example of symmetry or equivalence of interaction networks with respect to short-range signaling genes.

Among the configurations for (*N*,*H*) = (4,2) and through the approach presented in Methods, there are 405 908 genetic networks that reach point attractors, giving rise to 457 different organism (or tissue) patterns produced by 43 distinct gene patterns. Some patterns are very common, as they can be produced by many distinct networks, while other patterns result from very specific topologies. Ordering or ranking by decreasing frequency associates thus a rank to the patterns, resulting into the distribution *N*_1_(*r*) of a rank *r*. It has been reported to follow Zipf's law, *N*_1_(*r*)∝*a*(*b*+*r*)^−*γ*^, for both RNA folding [19] and feed-forward signaling nets [52]. In the present case, the observed distribution follows a power law *N*_1_(*r*)∝*r* ^−*γ*^, with *γ_t_* = 2.3 for tissue patterns frequency (Figure 3A) and *γ_g_* = 3.8 for gene patterns frequency (Figure 3B).

The system of local-genes and morphogenes as described above presents symmetry with respect to the latter (Figure 3C). The symmetry in the local genes is broken by the initial condition–first gene is active. Therefore, a significant majority of the interaction matrices have a symmetric pair that is equivalent in terms of the interactions and thus resultant expression pattern (457 tissue patterns reduce to 263 unique or non-degenerate patterns). However, in the present study we have addressed also the issue of evolution, for which the totality of possible networks has to be employed in order to allow for different evolutionary paths. Thus, we chose to maintain this degeneracy.

#### Stripe expression pattern

**Figure 4:**
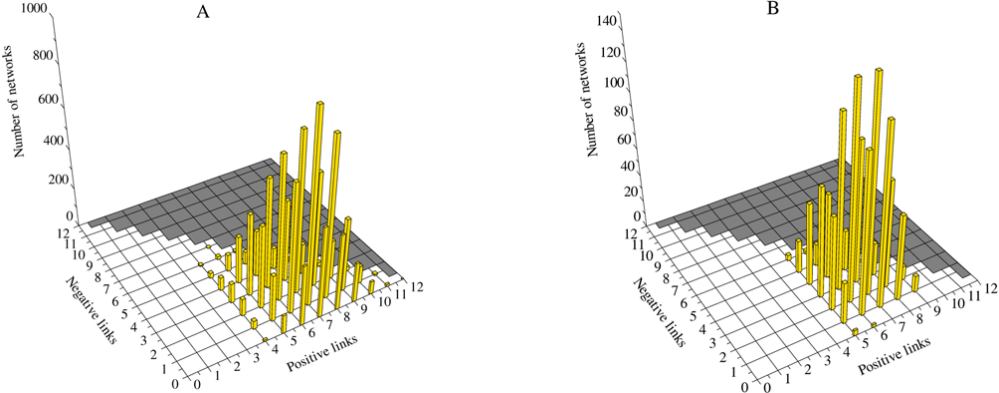
The distribution of positive and negative interactions. (A) The distribution of the 9753 networks that present activity in all genes (*A* = 1), and (B), the 1394-networks subset presenting stripes in at least one gene.

As mentioned before, we are looking for stable alternating active-inactive expression patterns of the genes, as some of the examples in Figure 3A and 3B. In the right panel of Figure 4 we represent the number of network configurations yielding stripe patterns. Here we use a two-dimensional parameter space defined by the number of positive (*L*_+_) and negative (*L*_−_) links of individual networks. Since we have *N* = 4 genetic elements, *N* ^2^−*H* ^2^ = 12 links are possible and thus *L*_+_+*L*_−_≤12 (gray area in 3D histogram figures). We remark that a certain number of positive interactions is necessary for the existence of the stripe expression pattern. As a first step, there is a threshold of *L*_+_ = 4 positive links required such that all genes are active in at least one cell (left panel of Figure 4), a requirement that is prior to that of the stripe pattern, as the latter cannot occur without the former.

#### Robustness

There is increasing evidence of the invariance of phenotypes under several types of perturbations [7],[35]. Several classes of biological robustness have been already defined in the context of genetics [60]. Intrinsically related to neutrality is the genetic robustness: the resilience of phenotypes with respect to genetic variation. There is also the notion of environmental robustness that refers to buffering against external environmental fluctuations. Related to both types of robustness, but on a higher hierarchical level, is the developmental robustness [60] defined through the robustness to internal micro-environmental fluctuations or stability under developmental noise.

In the present model, a form of developmental robustness measure is employed, defined through the percentage of perturbed expression experiments leading to the recovery of the same stable pattern. More precisely, in a serial manner and for the stable expression pattern, as we flip one by one the state of all genes in all cells, we determine if the stable expression pattern is recovered. Thus the robustness values belong to the interval [0,1], with step 1/(*N*×*C*), where *C* is the number of cells.

**Figure 5:**
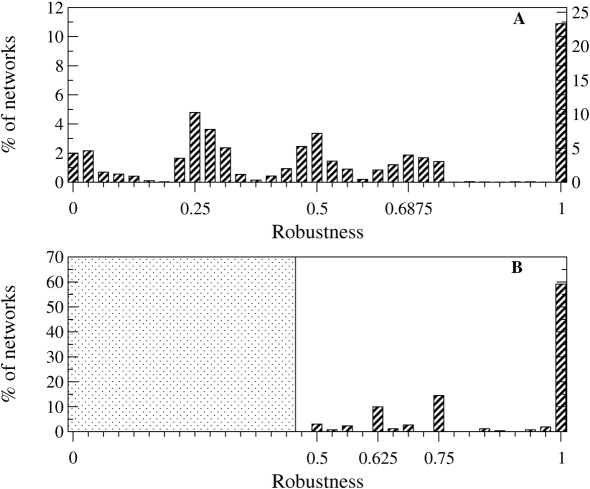
Robustness histogram for the networks producing non-null stable gene expression. (A) For a better visualization, two normalizations have been used: for the left ordinate, using the entire set of stable networks (405 908 networks), and for the right ordinate, using the non-null stable networks (189 658 networks). (B) The robustness histogram for the 524 networks producing all stripes (Entropy *H* = 1; eq. 7 ). The dotted area puts in evidence that there are no *H* = 1-networks with robustness *R*<0.48.

In Figure 5 we represent the robustness values of the non-null stable expression patterns produced by the interaction networks. We remark the non-uniformity of the distribution, with regions of forbidden robustness. The cell-cell communication engenders these classes of robustness, as the diffusible paracrine molecules define regions of cells of a characteristic inter-dependence.

**Figure 6:**
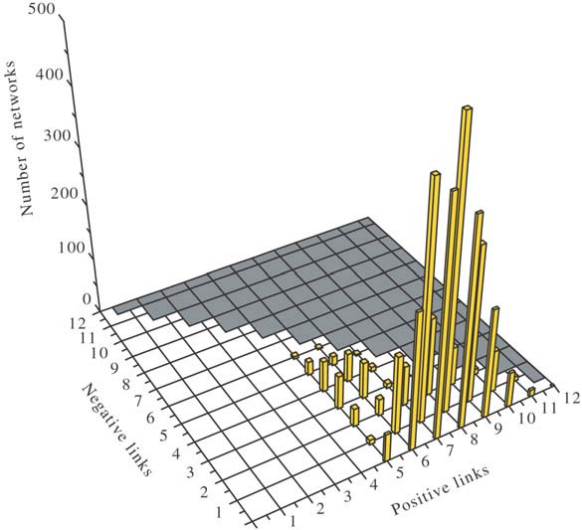
Robust networks. The 3409-networks entirely robust subset from left panel of Figure 4A (*A* = 1 and *R* = 1).

In addition, we have searched for an indication of a fundamental causal feature of the entirely robust networks. Among the non-null maximum robustness networks (forming the highest peak in Figure 5A), not all present activity in all genes. We noticed that non-null entirely robust networks exist with at least 3 positive interactions. However, we are interested again in functional networks, those leading to expression of all genes in at least one cell (left panel of Figure 4). Intuitively, at least 4 activation interactions are needed such that activity propagates to all genes, but it is interesting that one more link is generally needed to make such patterns robust (Figure 6).

#### Accessibility and adjacency

**Figure 7:**
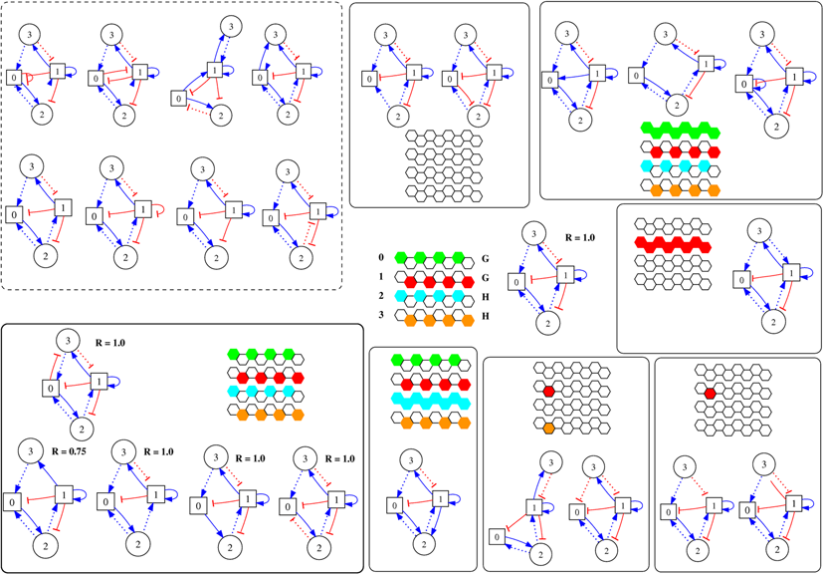
The 24 one-link neighbors of a robust network (center) producing stripes. The rectangles group together networks producing equal expression patterns. The upper left networks do not produce fixed-point patterns. The lower left rectangle includes those networks that have the same pattern as the central network. For these, the value of robustness is also indicated.

Through random mutations, organisms may wander in the phenotype space, maintaining the phenotype through neutral mutations, or changing it to better or worse phenotypes. The comparison depends generally on a fitness function or on its proximity to an environmentally-defined optimum (target) phenotype. In Figure 7 we show an example. Among the 24 (12 links×2 new states) neighbors in the genotype space of every given network, there are some that maintain the expression patterns. But these patterns may present the same or different value of the expression robustness. In this way, there may exist mutations that are neutral in the expression pattern, but showing more or less robustness. Relating to various definitions of robustness [60], one can see that our study combines two definitions of robustness, the mutational robustness (or neutrality) and the developmental robustness, as defined above. Thus, the evolutionary study presented in the following section intends to provide clues on the evolution of minimal developmental modules as well as to reveal pathways towards this goal, in the spirit of the elegant hypothesis [61]. More precisely, this issue of accessibility or adjacency of new, improved or equivalent phenotypes plays a central role in the evolution towards robust expression patterns in this simple model.

### Evolution

Robustness and evolution have been shown to be closely linked, even though there is no consensus on this correlation being entirely positive or rather a positive-negative trade-off [62],[63]. The developmental scheme has to be robust enough to guarantee a reliable organism but not too robust to impede evolutionary changes and thus improved adaptive solutions. In this direction, theoretical studies of gene networks can shed light on the mechanisms responsible for this trade-off. Such a task is difficult to assign to experimental approach but perfectly assignable to theoretical modeling, even though the inspiration and final results relate to the fossil record [64] and experimental work [65].

In this context and for the evolutionary part of our study, we have associated a fitness function weighting pattern complexity. The fitness function associated to a given phenotype is inspired in previous works on the RNA folding landscape [66] and it is:
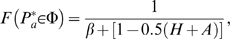
(6)with *β* = 0.01 and 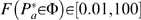, where the parameters *H* and *A* are the entropy and activity measure, respectively. Networks giving rise to unstable expression patterns are attributed a minimum fitness value, *F* = 0.01. The measure of activity, *A*, is defined as the fraction of the genes active in at least one cell. As we study the case (*N*,*H*) = (4,2), the activity *A* takes the values 0.0,0.25,0.75,1. The activity *A* is introduced in order to guarantee that all genes are used at least once through development. The entropy of the resultant gene expression is a measure of the heterogeneity of the pattern and is defined in terms of
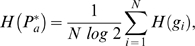
(7)where *H*(*g_i_*) is the (spatial) entropy of the *i*-th gene. Since only ON-OFF states are allowed, it reduces to
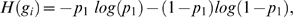
(8)being *p*_1_ the probability that *g_i_* takes the ON state, i.e. 
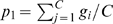
. As defined, *H*(*g_i_*) = 0 for a fully homogeneous pattern and *H*(*g_i_*) = *log* 2 for a pattern with equal number of ON and OFF states.

**Figure 8:**
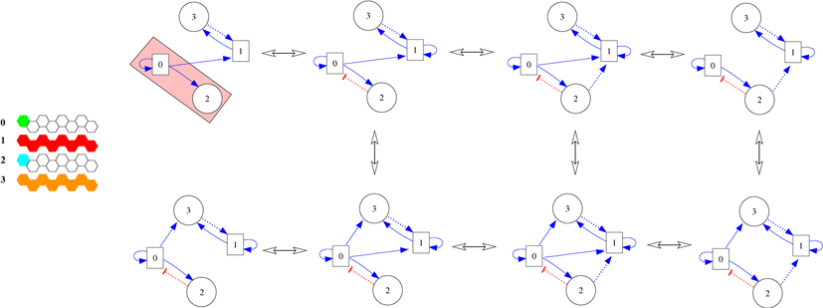
An example of neutrality in diversity. Several networks producing the same pattern of equal fitness value are accessible through mutations of one link. As a detail, all these network share the same value of robustness. Notice the conserved structure in all networks isolated in the reddish rectangle.

Having defined the fitness function, the entire fitness landscape for the case-study of *N* = 4 can be calculated. A glance at the fitness landscape shows that only through one-link mutations, a given expression pattern and/or fitness value can be maintained in long neutral paths. For illustrative purposes, we arbitrarily chose an example of such neutrality in diversity in Figure 8 by a path of one-link mutations maintaining the expression pattern (fitness) and robustness.

**Figure 9:**
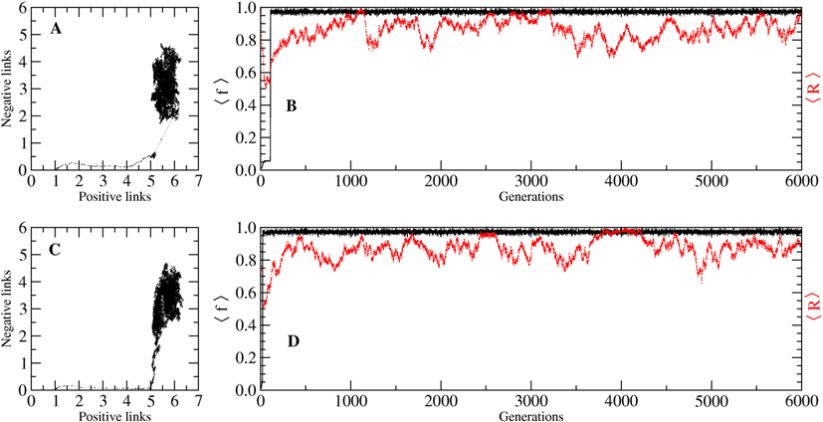
Examples of evolution experiments. (*A and C*) The evolution in the space of positive and negative links, and (*B and D*) the time evolution of the mean fitness (in black; 〈*f*〉 = *β*〈*F*〉, normalized such that 〈*f*〉∈[1,0]), and robustness (in red). A population of *N* = 500 networks was used with mutation rate *μ* = 0.01 per network.

In our evolutionary study, we have used a constant population model (*N* = 500 networks) of non-overlapping generations, with the individual networks replicating according to their fitness. By simulating the temporal evolution of this population initiated by identical networks of only one link, we have witnessed the increase in the average fitness of the population as more diverse patterns appear. A couple of examples of such evolutionary paths is shown in Figure 9. We display both the time evolution of the mean fitness (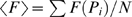) and robustness (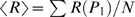), and the corresponding path in the (*L*_+_,*L*_−_) space. As a general trend in our evolutionary experiments, we have noticed that the population rapidly becomes dominated by stripe networks, constituting a stable almost-unitary fraction of the total population. It is interesting to remark that, even though the mean robustness varies, it fluctuates around a high value. This behavior is expected, as one can infer from the robustness distribution of the stripe networks (Figure 5B). Even so, it remains an important result, as it intrinsically relates high robustness with stripe patterns.

As a general characteristics for the evolutionary paths, we have noticed that all networks increasingly acquire positive interactions (Figure 9A and 9C) which provide an increase in diversity, and implicitly in the entropic measure *H*. The last steps prior to reaching the maximum fitness are characterized by the acquisition of negative regulatory interactions, stabilizing and diversifying the expression pattern.

**Figure 10:**
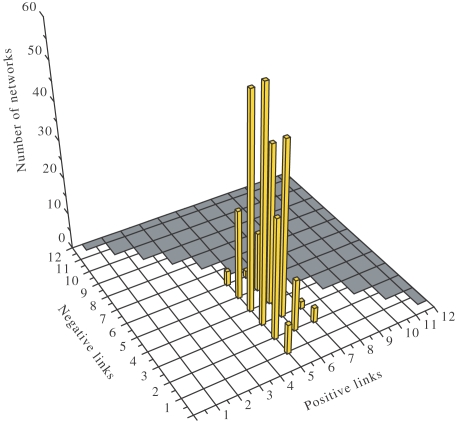
The fittest networks. The distribution of the 310 fittest networks of maximum robustness in the space of positive and negative links. The 3D view produces an apparent symmetry in the histogram's peaks that, at a more careful inspection, does not exist.

We wondered about the particularities of the networks of maximum fitness together with maximum robustness. First of all, there exist several such networks characterized by a proper balance between activating and inhibiting interactions (Figure 10). In average, this proper balance results to be *L*_+_/*L*_−_≈1, and ensures their robustness and the maximum diversity of expression pattern.

Interestingly enough, all these networks present the same expression pattern, a stripe pattern in all genes. We expected that maximum fitness networks could be of non-stripe pattern, as maximum diversity can be obtained through other patterns too (e.g. an all-active half plus an all-inactive half; similar to the example of gene pattern in Figure 3B). Unexpectedly, no other pattern of maximum diversity other than the all-stripes one exists among the stable patterns. This points to the fact that, in such a minimal pattern-formation module, there is a tight inter-dependence between the stripe-like pattern and high robustness values. This is supported by two issues. The first indication is related to the robustness distribution of the stripe networks (Figure 5B) where it can be seen that they are biased towards high robustness values, with more than 60% of them having maximum robustness. The second argument, as mentioned above, relates to the fact that non-stripe networks of maximum entropy do not exist among stable networks. Even though individual genes may be present in an organism in the form of all-active half and an all-inactive second half, these individual gene patterns do not combine into an *H* = 1 stable organism. In fact, we notice that such a gene pattern exists in stable organisms only in combinations with null gene pattern. Finally, in support of the tight relationship between robustness and stripe networks, the neurogenic network in *Drosophila* embryo has been shown to present such inter-dependence [48],[67], and we shall come back to this issue shortly.

Moreover, we remarked that all these robust stripe networks form a connected meta-graph or a neutral meta-graph, where connections imply one-link mutation. A relevant conclusion from this observation relates to the stability of the expression pattern against changes in the interaction rules. The robustness to the interaction rules relates to genetic robustness, in which gene knock-outs are contemplated. Such robustness has been observed for the developmental module that underlies the *ABC* model of floral organ specification in *A. thaliana* [53], consistent with an overall floral plan widely conserved among flowering plants. Similarly, structural alterations (gene knock-outs) of the neurogenic gene regulatory network in *Drosophila* appear to be well tolerated by the system from the point of view of the resultant gene expression [48].

In the general context of genome architecture, there is undeniable evidence of redundancy (or multiple backup circuits) [68] as a key element, though not unique, responsible for this structural robustness property. This type of robustness manifests itself by the resilience of circuit designs to the removal or loss of a given unit. In the relationship between robustness and modularity too, it is interesting to mention the distinction between redundancy and degeneracy [69], where degeneracy refers to different units performing a given function, while the redundancy relates to the presence of multiple identical copies of a unit. Several models have explored the role of evolution in driving the formation of backup circuits [70],[71], emphasizing the gene duplication processes as the primary dynamical building-block of innovation [72].

**Figure 11:**
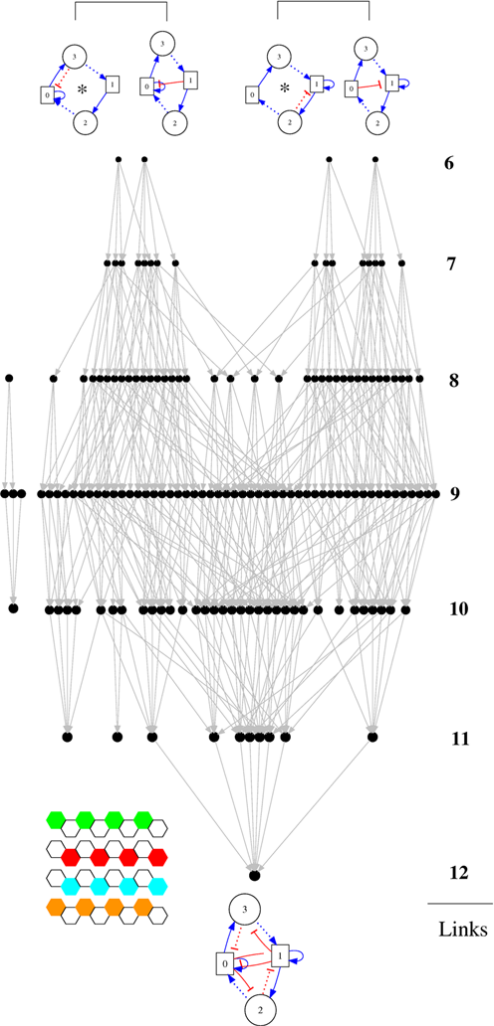
The inclusion directed meta-graph of the fittest distinct 155 networks. The nodes represent networks, and a network *A* is linked to a network *B* if one link has been added to the former to produce the latter. Naturally, the 155 symmetric equivalents of these networks have maximum robustness too. The minimal fittest networks are the upper networks grouped in pairs of identical trajectory towards the stable pattern. See text for details.

It is worth noticing that there exist several minimal networks at the root of all these particular best networks. By minimal we refer to the minimum number of genetic interactions leading to this robust fittest phenotype. For visualizing the relationship between them, we have represented all the fittest networks in the form of an inclusion directed meta-graph in Figure 11. Nodes represent networks, and we considered that network *A* is connected to network *B* if one link has been added to the network *A* to produce network *B*. As a detail, the size of the node is an indication of the number of constitutive interactions of the associated network. All these networks have in common the same gene expression pattern, a pattern characterized by stripe-like expression for all the genes (Figure 11).

**Figure 12:**
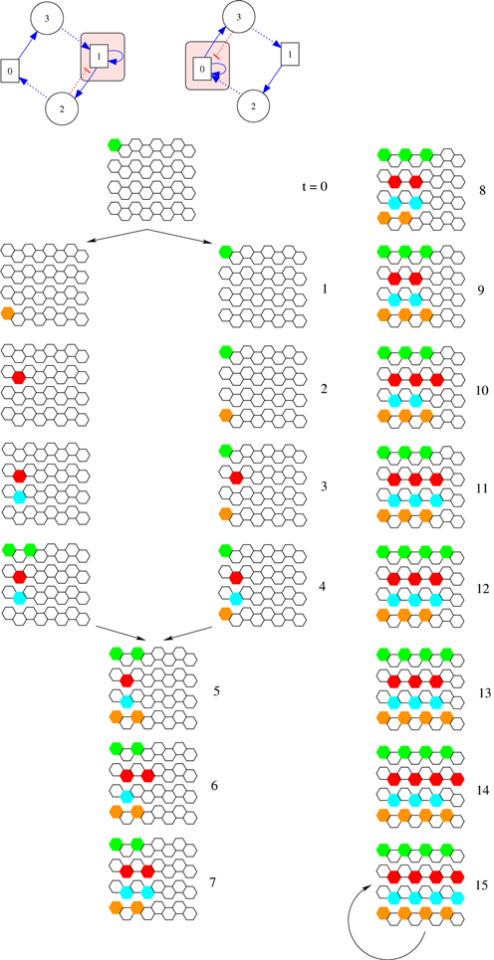
Two minimal networks and their trajectory towards the stable pattern, from the initial condition to the final stable pattern. In the colored boxes of the networks we emphasize the motif responsible for the stripe-like pattern. See text for details.

In addition to these symmetry considerations, we also noticed that pairs of these minimal networks (brackets in the upper part of Figure 11) share common construction of the stable expression pattern from the initial condition. For illustrative purpose, the steps necessary to reach the stable patterns have been drawn in Figure 12 for two minimal networks (networks indicated by an asterisk in Figure 11). One can identify a connection motif as the key element responsible for the robustness and diversity, a motif emphasized in Figure 12. By isolating this interactions in the colored boxes we emphasize also the fact that the inhibitory interaction can be provided either by a morphogen or a local gene (see the pairs under brackets in Figure 11).

The resultant robust configurations and the interaction motif recall a key process in pattern formation, especially in developing tissues: lateral inhibition with feedback [55]. Lateral inhibition refers to a type of cell-cell interaction in which a cell that adopts a particular fate inhibits its immediate neighbors from doing likewise. The modeling of the neurogenic genes *Notch* and *Delta*, and their associated trans-membrane proteins sheds light on the mechanism of amplification of differences between adjacent cells [73]. Moreover, it has been shown that for the neurogenic network in *Drosophila* embryo, the lateral inhibition buffers the expression pattern against perturbations (knock-outs) [48], resulting in a tight correlation between robustness and stripe-like pattern mentioned above.

### 2D Organisms

We have considered in the present study the one-dimensional organisms, as this approach provides a clarifying perspective on the basics of pattern formation in such minimal networks, and thus a faster identification of the underlying key features for robustness and diversity. Preliminary results on the 2D (*N*,*H*) = (4,2) case yield interesting comparisons with the 1D case. Among these, slightly more than 10% of the most-robust fittest 1D networks constitute the set of most-robust fittest networks (according to eq. 6) in the 2D case. In this context, although most of our qualitative trends are also observed, the number of non-null stable patterns is slightly reduced (189 658 in 1*D* compared to 165 856 in 2*D*). This decrease is consistent with the higher degrees of freedom allowed by the dimensional increase. It also opens the possibility of increased instability, and thus less robustness. In future works, we shall inquire on the necessary features of the interaction network leading to the maintenance of robustness and diversity independently of the spatial framework.

## Discussion

Embryonic development is a particular field in biology characterized by a constant feedback between theoretical analysis and experimental work. Even though experimentalists still remain cautious on the predictive power of the former, there have been important advances in clarifying the organizational principles of embryonic pattern formation [1],[3],[55],[58]. Restricting ourselves to studies on *Drosophila* development (even though the conclusions seem universal), extensive simulations have shown that topology constrains the possible behavior of a regulatory network [74]. Similar studies on plant development also support this conclusion [40]. Moreover, in the context of development and not only, a crucial relationship has been proved to exist between topology and robustness [39],[74].

It is thus apparent that under the requirements of a given phenotype, selection will ensure that increasingly stable networks of interactions evolve towards it. In this direction, developmental modules appear to play the major organizing role. These kernels of the entire developmental genetic network perform distinct regulatory functions and constitute information-processing units in the correct and precise unfolding process of development [75]–[77]. Thus, two of the central key topics of developmental biology are the evolution and robustness of patterning mechanisms, and the still unsettled relationship between them.

In this context, we have studied small epigenetic networks that could behave evolutionarily as minimal modules capable of producing a stripe expression pattern similar to those common in early embryonic development. In the present approach the minimal number of genes capable of producing such an expression pattern is *N* = 4, number that allows an exhaustive analysis of the genotypic space. Considering both topological and robustness issues, we have determined the space of expression patterns produced by such module using a dynamical modeling inspired from previous related studies of Boolean and continuous models [50],[52],[78],[79]. Among all possible expression patterns, we have identified those presenting enhanced reliability in maintaining their expression through perturbations. From performing evolutionary experiments (Figure 9), we can conclude that the paths towards the most robust and diverse expression pattern are short. In other words, the optimal modules are rapidly encountered in the landscape.

We find necessary a comparison between the above-mentioned continuous models and the currently employed discrete approach. The former works are related to a different class of assumptions, both on the dynamical side (namely, Michaelis-Menten kinetic description of gene-gene interactions) and in the type of questions being considered (namely, a statistical study of the parameter space and network structure). In these works, search algorithms explored extended regions of the parameter space and, once a pattern-forming network was found, a network reduction process was applied in order to find minimal modules. The leading mechanisms pervading the formation of stripes cannot be directly compared with our study (where the equivalent nonlinearities would be of higher order, Hill-like class). Moreover, we have concentrated here on a well-defined, small-sized network such that the calculation of the entire space of possibilities could be feasible. Exploring the landscape structure in such a systematic way would be much more difficult (if possible at all) under the continuous approximation, and thus our conclusions need to be restricted to the discrete level. Nevertheless, we consider that a direct comparison of results between continuous and discrete models requires a detailed dedicated study. At least in the segment polarity network in *Drosophila*, there is general agreement between continuous and discrete models. That is, comparison has been conducted between approaches associated to a given system and thus characterized by similar assumptions. A general comparison of capabilities and limitations of discrete versus continuous models has not been addressed, as far as we know, and it is thus an important open question.

Here the analysis of the most robust modules uncovered a set of networks, all forming a meta-graph where links are one-point mutations between networks. The existence of this meta-graph is an indication of structural robustness of such networks, as many mutations can be neutral. Also associated to this set, there exist certain minimal networks responsible for robustness and diversity, and many additional interactions provide a back-up mechanism or alternative pathways. The generic properties of the optimal modules indicate thus that lateral inhibition is likely to be a generic form of creating ON-OFF spatial patterns, although the exact structure of the generating module might differ, given the observed neutrality. Future work will explore how these modules might emerge and evolve within larger gene regulatory webs, the underlying phylogenetic patterns as well as the impact of network topology on evolvability and developmental plasticity.

## Methods

The equations determining the evolution of genes' state in time are:
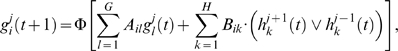
where ∨ is the “OR” function. Similarly, genes coding for short-range signaling molecules receive inputs only from the first set,
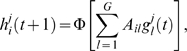
with specific equations at the boundaries reading:
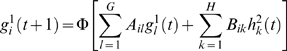


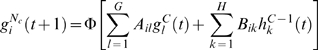



The function Φ(*x*) is a threshold function, i.e. Φ(*x*) = 1 if *x*>0 and zero (inactive) otherwise. Given the initial condition and after transient time *T* ( = N*C, with *C* the number of cells) time steps, we check on the stability of the resultant pattern, considering only the fixed-point attractors and not the oscillatory ones.We consider such a relatively short transient time as relevant to the evolutionary studies that we shall introduce in the following section.

As defined, the phenotype in our model is given by the steady state defined by the *N*×*C* matrix **P** ^*^ given by:
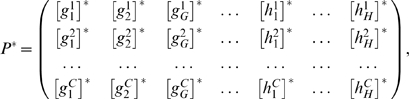
where 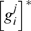 and 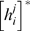 indicate the stationary values of each regulatory element after the transient. With our previous definitions, we can properly define the mapping
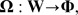
where for each genotype *W_a_* = (*A_ij_*, *B_kl_*)∈**W**, we have a phenotype 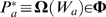. The distance between two genotypes, *W_a_* and *W_b_* is defined by
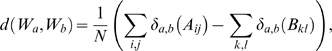
(9)where *δ_a_*_,*b*_(*A_ij_*) = 0 if 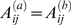, and it is 1 otherwise. If *d*(*W_a_*,*W_b_*) = 1, the networks *W_a_* and *W_b_* are connected in a meta-graph (see Figure 11). The Python code developed for the calculation of the fitness landscape and for the evolution experiments is available as Protocol S1. The dataset corresponding to the landscape of the study case (*N,H*)  =  (4, 2) is also hosted online as Dataset S1.

## Supporting Information

Dataset S1 The dataset includes the results of the exhaustive simulations of all stable networks of 4 genes, as detailed in the manuscript.
(1.70 MB ZIP)

Protocol S1 The files consist of the Python codes developed for and employed in the simulations of the gene expression profiles for the case of 4-gene networks detailed in the manuscript.
(0.02 MB PDF)
