# Supplementary material for: Neutrality and Robustness in Evo-Devo: Emergence of Lateral Inhibition
Source: PLoS Comput Biol. 2008 Nov 21;4(11):e1000226. doi: 10.1371/journal.pcbi.1000226 (PMC2577890; doi:10.1371/journal.pcbi.1000226)
Supplement: Protocol S1 — The files consist of the Python codes developed for and employed in the simulations of the gene expression profiles for the case of 4-gene networks detailed in the manuscript. (0.02 MB PDF) [file pcbi.1000226.s002.pdf]

```
## (c) 2008, Andreea Munteanu
## Initiated October 2007, Santa Fe Institute

from numpy import *
from gene_net import *
import pickle
from RandomArray import *
from time import time, asctime
import socket



def save2file( _file, Patterns ):
    out_file = open(_file, 'w')
    pickle.dump(Patterns, out_file)
    out_file.close()


########################################################################
NumCells = 8
Width = 4
stoch_runs = 1 #will not be used.

t_start= time()

N = int( sys.argv[1] )
H = int( sys.argv[2] )
Width = int (sys.argv[3] )
id1 = int( sys.argv[4] )
id2 = int( sys.argv[5] )
part_idx = sys.argv[6]

if Width > 1 :
    out_filename = "2DHEX_N%d_H%d" % (N, H)
else:
    out_filename = "1D_N%d_H%d" % (N, H)
if len(sys.argv) > 5 :
   out_filename = out_filename + ".Part" + part_idx

log_filename = out_filename + ".log"
log_file = open(log_filename, 'w')
log_file.write ("Command: %s\n" % sys.argv )
log_file.write ("On host: %s\n" % socket.gethostname() )
log_file.write("Launched: %s\n" % asctime())
log_file.close()


G = N − H
OutputPatterns = {}

Max_id = pow( 3, N*N − H*H)
id2 = min( id2, Max_id )
for net_id in range ( id1, id2 ):

    W = generate_topology ( N, H, net_id )
    assert ( is_restricted ( W, G, H ))

    avg_organism = run_topology ( W, G , H, Width, NumCells )

    genes_stability = organism_stability ( avg_organism )

    if alltrue(equal( genes_stability, True )):
        string_pattern =  organism_to_string ( avg_organism)
    else:
        string_pattern = "U"

    if string_pattern not in OutputPatterns.keys():
        OutputPatterns[string_pattern] = []
    OutputPatterns[string_pattern].append( net_id )
```



```
    if ( net_id % 1000 == 0):
        save2file ( out_filename, OutputPatterns )

save2file ( out_filename, OutputPatterns )
t_end = time()

log_file = open(log_filename, 'a')
log_file.write("Duration: %d" % int(t_end − t_start) )
log_file.close()
```

```
## (c) 2008, Andreea Munteanu
## Initiated October 2007, Santa Fe Institute

import numpy
import random
from numpy import alltrue, sometrue, equal
from numpy import concatenate, ones, zeros, dot, array, array2string
from math import fabs, ceil, modf

noise_flag = False


def set_noise_flag ( _bool ):
    global noise_flag
    noise_flag = _bool


def to_base3( n ):
    digit = [str( n%3 )]
    if n < 3:
        return digit
    else:
        return to_base3( n / 3) + digit


############## UTILS:

#Topology:

def new_int_weights( n ):
    return numpy.random.randint( −1, 2, ( n, n ) )
    #g+h random arrays of g+h int elements.


def restrict_topology ( weights, g, h ):
    #hormones do not affect hormones.
    for i in range( g+h ):
        for j in range ( g+h ):
            if i >= g and j >= g:
                weights[i][j] = 0


def is_restricted( weights, g, h ):
    for i in range( g+h ):
        for j in range ( g+h ):
            if i >= g and j >= g and weights[i][j] != 0:
                return False
    return True


def generate_topology ( N, H, net_id): #needs refactoring
    G = N − H
    State = [ 0, −1, 1]
    interactions3 = to_base3 ( net_id )

    #base3_to_gene_interactions
    interactions = zeros ( len ( interactions3))
    for i in range( len(interactions3)):
        interactions[i] = State[int(interactions3[i])]

    #Restrict the topology: interactions to weights
    W = zeros ( (N,N) )
    k = len (interactions) − 1
    for i in range ( N − 1, −1, −1 ):
        for j in range ( N −1, −1, −1):
            if i < G or j < G:
                W[i,j] = interactions[k]
                k −= 1
            if k < 0: break
```



```
            if k < 0: break
    return W


def get_net_id ( W, N, H ):
    State={}
    State[0] = 0
    State[−1] = 1
    State[1] = 2
    _elements = N*N−H*H
    interactions = zeros (_elements)
    k = len (interactions) − 1
    for i in range ( N − 1, −1, −1 ):
        for j in range ( N −1, −1, −1):
            if i < N−H or j < N−H:
                interactions[k] = State[W[i,j]]
                k −= 1
    index = 0
    for i in range(_elements):
        index += interactions[i]*pow(3,_elements − i − 1)
    return int(index)


def check_networks_equivalence (net_id1, net_id2, N, H, gene1, gene2):
    W1 = generate_topology ( N, H, int(net_id1))
    W2 = generate_topology ( N, H, int(net_id2))

    if ( array2string(W1[0:N−H, 0:N−H]) == array2string(W2[0:N−H, 0:N−H]) ):
        if (array2string(W1[gene1,:]) == array2string(W2[gene2,:])
            and array2string(W1[gene2,:]) == array2string(W2[gene1,:]) ):
            if ( array2string(W1[:,gene1]) == array2string(W2[:,gene2]) and
                 array2string(W1[:,gene2]) == array2string(W2[:,gene1]) ):
                return True
    return False


def neighbors_of ( net, N, H ):
    Neighbors = []
    W = generate_topology ( N, H, net )
    for i in range(N):
        for j in range(N):
            if i < N−H or j < N−H:
                States = [0,−1,1]
                _state = W[i][j]
                States.remove ( W[i][j] )
                while ( len(States) > 0 ):
                    W[i][j] = States[0]
                    net_id = get_net_id ( W, N, H )
                    Neighbors.append( net_id )
                    States.remove ( W[i][j] )
                W[i][j] = _state
    net_id = get_net_id ( W, N, H )
    assert (net_id == net)
    return Neighbors


def networks_distance ( W1, W2, N ):
    distance = 0
    assert ( len(W1) == len(W2))
    for i in range( N ):
        for j in range ( N ):
            if  W1[i][j] != W2[i][j]:
                distance += 1
    return distance


def networks_distance_id ( net_id1, net_id2, N, H ):
    W1 = generate_topology ( N, H, net_id1)
    W2 = generate_topology ( N, H, net_id2)
```

```
    return networks_distance(W1,W2, N)


def organisms_distance ( o1, o2 ):
    assert ( (len( o1 ) == len( o2) and ( len(o1[0]) == len(o2[0]) ) ),
            'Compare organisms of diff lenghts! %d %d ' % (len( o1 ), len( o2 ) ))
    width = len(o1)
    num_cells = len( o1[0])
    num_genes = len(o1[0][0])
    distance = zeros ( (width, num_genes) )
    for j in range ( width  ):
        for i in range ( num_genes ):
            for c in range ( num_cells ):
                g_reporter_1 = o1[j][c][i]
                g_reporter_2 = o2[j][c][i]
                diff = (g_reporter_1 − g_reporter_2)
                distance[j][i] += diff * diff
            distance[j][i] /= num_cells
    return distance

## Cell states:
def new_cell_state( n ):
    return array( [0] * n )


## Tissue / Organism:
def new_organism( num_cells, n ):
    return [ rnd_state( n ) for i in range(num_cells) ]


def crafted_organism( mtx ):
    return [ [array(l) for l in mtx] ]


def crafted_stripes ( num_cells, all_genes, width = 1 ):
    pattern = zeros ( all_genes )
    organism = [ [ pattern for l in range(num_cells) ] ]
    for i in range(width):
        organism[i][::2] = [ ones( all_genes ) for l in range( int(ceil(num_cell
s/2.)) ) ]
    return organism


def print_organism ( o ):
    for c in o:
        print_cell_state ( c)


def equal_organisms( o1, o2 ):
    if equal(o1,o2).all() :
        return True
    else:
        return False


def copy ( organism ):
    result = list([])
    for cell in organism:
        result.append ( array(list(cell)) )
    return result


#String utils:
def netID_to_string ( net_id, N, H ):
    W = generate_topology ( N, H, net_id)
    return top_to_string( W )
```



```
def top_to_string( W ):
    _topology = ''
    N = len( W )
    for i in range( N ):
        for j in range( N ):
            if W[i][j] < 0:
                _topology += 'I'
            elif W[i][j] > 0:
                _topology += 'A'
            elif W[i][j] == 0:
                _topology += '−'
            else:
                _topology += 'Non integer values'
    assert ( len( _topology ) == N*N )
    return _topology


def top_to_array ( top_string ):
    N = len( top_string)
    assert ( N == (G+H)*(G+H) ), \
           'Topology size %d %d!' % (N, (G+H)*(G+H) )
    W = zeros( (G+H)*(G+H))
    i = 0
    for s in top_string:
        if s == 'A':
            W[i] = 1
        elif s == 'I':
            W[i] = −1
        i += 1
    W = resize(W,(G+H,G+H))
    return W


def gene_patt_to_string( o, g_id ):
    _pattern= ''
    for c in range( len(o) ):
        _pattern += `int( o[c][g_id] )`
    return _pattern


def organism_to_string ( o ):
    _pattern = ''
    width = len(o)
    num_cells = len(o[0])
    num_genes = len(o[0][0])
    for i in range(width ):
        for g in range( num_genes ):
            for c in range( num_cells):
                #_pattern += `int( o[c][g] )`
                _pattern += str(int( o[i][c][g] ))
            _pattern += '|'
        _pattern += '@'
    return _pattern[:−1]


def string_to_organism ( organism_str, N, Cells ):
    result = []
    tmp = [zeros( (N) ) for i in range( Cells )]
    _id = 0
    _w = 0
    while _id < len(organism_str) :
        if organism_str[_id] == '@':
            result.append(tmp)
            _w += 1
            _id += 1
        else:
            g = 0
            while g < N:
                if organism_str[_id] != '|':
```

```
                tmp[ (_id − (N * Cells +N + 1)*_w − g)%Cells ][g] =  \
                    int(organism_str[_id])
            else:
                g += 1
            _id += 1
    result.append(tmp)
    return result


def string_distance (s1, s2):
    distance = 0
    assert ( len(s1) == len(s2))
    for i in range(len(s1)):
        if s1[i] != '|':
            distance += fabs( int(s1[i]) − int(s2[i]) )
    return distance



#######################################################
##### STABILITY:
def gene_is_stable ( organism, g_id ):
    width = len(organism)
    for j in range(width):
        for cell in organism[j]:
            ( reminder, integer ) = modf( cell[g_id] )
            if reminder > 0:
                return False
    return True


def organism_stability ( organism ): #Stability of all genes is considered.
    width = len(organism)
    num_cells = len(organism[0])
    num_genes = len( organism[0][0] )
    Stability = [ True ] * num_genes
    for g in range( num_genes ):
        Stability[g] = gene_is_stable ( organism, g )

    return Stability



##### ROBUSTNESS:
def network_organism_robustness ( weights, pattern, N, H, NumCells ):
    #the average of gene robustness:
    # #the response is in (0,1) with step 1./(N*NumCells)
    result = network_gene_robustness ( weights, pattern, N, H, NumCells )
    NetRobustness = 0.0
    for R in result:
        NetRobustness += R
    NetRobustness /= N
    return NetRobustness


def gene_robustness ( o , W, G, H, g_idx):
    #perturb gene g_idx serially in all cells
    #and check if the final organism is the same
    #the response is in (0,1) with step 1./(NumCells*Width).
    initial_pattern =  organism_to_string ( o )
    robustness = 0
    width = len(o)
    NumCells = len(o[0])
    for cell_y in range(width) :
        for cell_x in range(NumCells):
            perturbed_pattern = \
                perturb_gene(copy(o), W, G, H, g_idx, cell_x, cell_y )
            if perturbed_pattern == initial_pattern :
                robustness += 1
```



```
    robustness /=   float(NumCells * width )
    return robustness


def network_gene_robustness ( weights, pattern, N, H, NumCells ):
    result = []
    organism = string_to_organism ( pattern, N, NumCells )
    for g_idx in range(N):
        robustness = gene_robustness (copy(organism), weights, N−H, H, g_idx)
        result.append ( robustness )
    return result


def perturb_gene ( o, W, G, H, g_idx, cell_x, cell_y = 0):
    o[cell_y][cell_x][g_idx] = 1 − o[cell_y][cell_x][g_idx]
    for i in range(15):
        o = next_organism( o, W, G, H )
    string_pattern =  organism_to_string ( o )
    return string_pattern



##### TIME PROPRESSION:
def mixed_cell_state( middle, neigh,  g ): #Hormones mixed states.
    ors = OR_function (neigh, g )
    return concatenate( (middle[0:g], ors) )
    #Genes remain unchanged. Have no mixed states.


def OR_function (neigh, g ):
    ors = neigh[0][g:]
    for i in range(1, len(neigh)):
        ors = map(lambda a,b: a or b, ors, neigh[i][g:] )
    return ors


def cell_neighbors ( o, x, y , system ):
    #x denotes horizontal index, cell index
    #y denotes vertical index, width index
    neigh = []
    width = len(o)
    length = len(o[0])
    if system == 'LINE':
        assert ( width == 1 )
        if x == 0:
            neigh = [ o[0][1] ]
        elif x == length − 1:
            neigh = [ o[0][−2] ]
        else:
            neigh = [ o[0][x−1], o[0][x+1] ]
    #######
    if system == 'HEX':
        if y%2 != 0: #even row
            shift = 0
        else: #odd row
            shift = −1

        #periodic vertical boundary:
        y_lower = (y + 1) % width
        y_upper = (width + y − 1) % width
        if x == 0:
            neigh = [ o[y][x+1], o[y_lower][x+1+shift], o[y_upper][x+1+shift] ]
            if x+shift >= 0:
                neigh.append ( o[y_lower][x+shift] )
                neigh.append ( o[y_upper][x+shift] )

        elif x == length − 1:
            neigh = [ o[y][x−1], o[y_lower][x+shift], o[y_upper][x+shift] ]
            if x+1+shift < length:
```

```
                neigh.append (o[y_lower][x+1+shift] )
                neigh.append (o[y_upper][x+1+shift] )
        else:
            neigh = [ o[y][x−1], o[y][x+1], \
                      o[y_lower][x+shift], o[y_lower][x+1+shift],\
                      o[y_upper][x+shift], o[y_upper][x+1+shift] ]
    return neigh



def next_cell_state( cell_state, weights):
    if 2 in cell_state :
        return cell_state
    else:
        sums = dot( cell_state, weights ) # Matrix dot product
        ( reminder, integer ) = modf( fabs (sum(sum ( weights ))) )
        if reminder > 0:
            return array([ int(int( s + int(noise_flag)*random.random() > 0) )
                           for s in sums ])
        else:
            return array([int(int( s+int(noise_flag)*random.randint(−1,1) > 0))
                           for s in sums ])

#
def initial_organism ( num_cells, n, width = 1):
    o = []
    for i in range(width):
        o.append([ zeros( (n) ) for j in range(num_cells) ])
        o[i][0][0] =1
    return o


def next_organism( o, weights, g, h ):
    result = []
    width = len(o)
    length = len(o[0])
    assert ( length > width )
    # first
    if width == 1:
        system = ’LINE’
    else: #extendable to other neighborhoods
        system = ’HEX’
    for y in range( width ) :
        line = []
        for x in range(length):
            mx = mixed_cell_state( o[y][x], cell_neighbors(o, x, y, system ), g)
            line.append( next_cell_state( mx, weights ))
        result.append ( line )
    return result


def average_organism ( o, weights, g, h, steps ):
    width = len(o)
    num_cells = len( o[0])
    avg_organism = [ ]
    for i in range(width):
        avg_organism.append( [zeros( (g + h) ) for i in range( num_cells )] )

    for r in range( steps ):
        o = next_organism( o, weights, g, h )
        for j in range(width):
            for i in range( num_cells ):
                avg_organism[j][i] = avg_organism[j][i] + o[j][i]
    for j in range(width):
        for i in range( num_cells ):
            avg_organism[j][i] = avg_organism[j][i]/float(steps)
    return avg_organism
```



```
def after_transient_organism ( o, weights, g, h ):
    num_cells = len( o[0] )
    width = len(o)
    time_steps = 10 * num_cells * width
    for r in range( time_steps ): #Transient.
        tmp_o = o
        o = next_organism( o, weights, g, h )
        if equal(o,tmp_o).all():
            break
    return o


def get_pattern ( o, weights, g, h ):
    o = after_transient_organism ( o, weights, g, h )
    check_steps =  len(o[0])
    return average_organism ( o, weights, g, h, check_steps )


#
def run_topology ( W, G, H, w , NumCells ):
    _output_pattern = []
    for j in range( w ):
        _output_pattern.append( [ zeros( (G + H) ) for i in range( NumCells )])

    iterations = 1
    if noise_flag:
        iterations = 100


    for i in range ( iterations ):
        o = initial_organism ( NumCells, G + H, w) #Random initial condition.
        pattern = get_pattern (  o, W, G, H )

        for j in range(w):
            for c in range( NumCells ):
                _output_pattern[j][c] = pattern[j][c] + _output_pattern[j][c]
    for j in range(w):
        for c in range( NumCells):
            _output_pattern[j][c] /= iterations
    return _output_pattern


##### EVOLUTION:
def mutate_topology ( net_id, N, H ):
    States = [ 0, −1, 1]
    G = zeros((N,N))
    indexes = N*N − H*H
    idx = random.randint (0, indexes−1)
    W = generate_topology ( N, H, net_id)
    _it = 0
    _found = False
    for i in range(N):
        for j in range(N):
            if i < N−H or j < N−H:
                if _it == idx:
                    _found = True
                    break
                else:
                    _it += 1
        if _found == True:
            break

    G = generate_topology ( N, H, net_id)
    States.remove(G[i][j])

    idx_state = random.randint (0, len(States)−1 )
    G[i][j] = States[idx_state]
    assert (G[i][j] != W[i][j] )
    change = (W[i][j], G[i][j] )
```

```
    #this is just to make sure now (case (4,2).
    assert (G[2][2] == 0 and G[2][3] == 0  and \
            G[3][2] == 0 and G[3][3] == 0)
    m_net_id = get_net_id ( G, N, H)
    assert ( networks_distance_id ( m_net_id, net_id, 4, 2 ) == 1 )
    return m_net_id, change
```

```
## (c) 2008, Andreea Munteanu
## Initiated October 2007, Santa Fe Institute

from gene_net import *
from math import fabs
import unittest


def check_evol( o, W, lst ):
    for l in lst:
        o = next_organism( o, W, 1, 1 )
        m1 = crafted_organism( l )
        if not equal_organisms( o, m1 ):
            return False
    return True


class TestGeneNet1(unittest.TestCase):
    def setUp(self):
        pass
    def tearDown(self):
        pass

    def test_mixed_state(self):
        s1 = mixed_cell_state( array([0,0]), [ [], array([0,1])], 1)
        self.assert_( s1[0] == 0 and s1[1] == 1)

        s2 = mixed_cell_state( array([0,1,1]), [ [], array([0,1,1]) ] , 1)
        self.assert_( s2[0] == 0 and s2[1] == 1 and s2[2] == 1)

        neigh = [ [1,1,0,0], [1,1,1,0],[1,1,1,0], [1,1,0,0], [1,1,1,0] , [1,1,1,
0] ]
        s3 = mixed_cell_state( array([0,1,0,0]), neigh, 2)
        self.assert_( (s3[0] == 0 and s3[1] == 1 and s3[2] == 1 and s3[3] == 0),
 s3 )

    def test_next_state(self):
        W = array([[1,1],[−1,0]])
        state = [1,1]
        s1 = next_cell_state(state, W )
        self.assert_( s1[0] == 0 and s1[1] == 1 ,
                      'Next State: %s' % repr(s1) )



    def test_state_sequence(self):
        W = array([[0,1],[1,0]])
        o = crafted_organism( [[1,0],[0,0],[0,0],[0,0],[0,0]] )

        self.assert_( check_evol( o, W, [ [[0,1],[0,0],[0,0],[0,0],[0,0]],
                                          [[0,0],[1,0],[0,0],[0,0],[0,0]],
                                          [[0,0],[0,1],[0,0],[0,0],[0,0]],
                                          [[1,0],[0,0],[1,0],[0,0],[0,0]] ] )
                      )
    def test_string (self):
        o = crafted_organism( [[1,0],[1,0],[0,1],[0,0],[0,1]] )
        o_string = organism_to_string ( o )
        o_string_test = "11000|00101|"
        self.assert_( o_string == o_string_test,
                      'The organisms are: %s %s ' % (o_string, o_string_test) )


    def test_distance(self):
        g = 1
        h = 1

        o3 = crafted_stripes ( 5, 2 )
        o1 = crafted_organism( [[1,0],[1,1],[1,0],[1,1],[1,0]] )
        o2 = crafted_organism( [[1,0],[0,1],[1,0],[0,1],[1,0]] )
```



```
        D1 = organisms_distance ( o1, o2 )
        D2 = organisms_distance ( o3, o2 )
        self.assert_( sometrue(equal( D1, 0 )),
                      'Distance: %s' % repr( D1 ) )
        self.assert_( sometrue(equal( D1, 0 )) and sometrue(equal( D2, 1 )),
                      'Distance: %s' % repr( D2 ) )


    def test_pattern(self):
        g = 1
        h = 1
        W = array([[1,1],[1,0]])
        o = initial_organism ( 5, 2)
        target = crafted_organism( [[1,1],[1,1],[1,1],[1,1],[1,1]] )
        pattern = get_pattern (o, W, g, h )

        #W = generate_topology(4,2,41011)
        #o = initial_organism(5,4)
        #pattern = get_pattern (o, W, 2, 2 )
        #target = crafted_organism( [[1,0],[0,1],[1,0],[0,1],[1,0]] )
        self.assert_( equal_organisms ( target, pattern), pattern  )

    def test_hormones_interaction(self):
        g, h = 3,4
        for i in range(30):
            W = new_int_weights ( g + h )
            restrict_topology ( W, g, h )
            H = W[g:,g:]
            self.assert_( alltrue(equal(H,0)) )

    def test_stability(self):
        g = 1
        h = 1
        W = array([[1,1],[1,1]])
        o = initial_organism ( 5, 2)
        pattern = get_pattern (o, W, g, h )

        Stability = organism_stability ( pattern )
        self.assert_( alltrue(equal (Stability, True)),
                      'Stability 1: %s %s ' %  (repr(Stability), repr(pattern)) )


        W = array([[0,1],[1,0]])
        o = crafted_organism( [[1,0],[0,0],[0,0],[0,0],[0,0]] )
        pattern = get_pattern (o, W, 1, 1 )

        Stability = organism_stability ( pattern )
        self.assert_( sometrue(equal(Stability, False)),
                      'Stability 2: %s' % repr( Stability ) )

    def test_organism_operations(self):
        g,h = 1,1
        o = crafted_organism([ [1,0],[0,1],[1,0],[0,1],[1,0]] )
        cells = len(o[0])
        o1 = copy(o)
        self.assert_( equal_organisms ( o, o1 ) )
        o1[0][1][1] = 1 − o1[0][1][1]
        self.assert_( not equal_organisms ( o, o1 ) )
        o[0][1][1] = 1 − o[0][1][1]
        self.assert_( equal_organisms ( o, o1 ) )
        o_str = organism_to_string ( o )
        o2 = string_to_organism ( o_str, g+h, cells)
        self.assert_( equal_organisms ( o, o2 ), o2)


    def test_topology_equivalence ( self):
        net_id1 = 41010
        net_id2 = 15122
```

```
        net_id3 = 41038
        self.assert_( check_networks_equivalence ( net_id1, net_id2, 4, 2 , 2, 3
) )
        self.assert_( not check_networks_equivalence ( net_id1, net_id3, 4, 2 ,
2, 3) )


    def test_topology_robustness (self):
        net_id = 41011
        N = 4
        H = 2
        Cells = 4
        width = 1
        W = generate_topology ( N, H, int(net_id) )
        avg_organism = run_topology ( W, H , N−H, width, Cells )
        genes_stability = organism_stability ( avg_organism )
        _pattern = ’’
        if alltrue(equal( genes_stability, True )):
            _pattern =  organism_to_string ( avg_organism)
        assert (_pattern == "1010|0101|1010|0101|")

        organism = string_to_organism ( _pattern, N, Cells )
        NetRobustness = 0.0
        R_genes = network_gene_robustness ( W, _pattern, N, H, Cells )
        assert ( len(R_genes) == N )
        R_net = sum(R_genes)/float(N)
        self.assert_( R_net == 1.0 )

    def test_net_index (self):
        net_id = 41010
        W = generate_topology ( 4, 2, int(net_id) )
        net_id1 = get_net_id ( W, 4, 2 )
        self.assert_( net_id == net_id1, ’Ids: %d %d’ % (net_id, net_id1))



if __name__ == ’__main__’:
    unittest.main()
```
